# Nerve Growth Factor Enhances Tooth Mechanical Hyperalgesia Through C-C Chemokine Ligand 19 in Rats

**DOI:** 10.3389/fneur.2021.540660

**Published:** 2021-06-01

**Authors:** Rui Guo, Yiyin Chen, Lu Liu, Jing Wen, Hong Yang, Yafen Zhu, Meiya Gao, Hengyan Liang, Wenli Lai, Hu Long

**Affiliations:** ^1^State Key Laboratory of Oral Diseases and National Clinical Research Center for Oral Diseases and Department of Orthodontics, West China Hospital of Stomatology, Sichuan University, Chengdu, China; ^2^Beijing Stomatological Hospital, School of Stomatology, Capital Medical University, Beijing, China

**Keywords:** nerve growth factor, tooth movement pain, C-C chemokine ligand 19, trigeminal ganglion, rat

## Abstract

The nerve growth factor (NGF) plays an important role in the regulation of neuropathic pain. It has been demonstrated that calcitonin gene-related peptide (CGRP), a well-known contributor to neurogenic inflammation, increases neuroinflammatory pain induced by NGF. The inflammatory mediator that NGF most strongly induces is C-C chemokine ligand 19 (CCL19), which can recruit inflammatory cells by binding to the receptor CCR7 followed by promoting the response of neuroinflammation. However, the regulatory mechanism of NGF and CCL19 in tooth movement orofacial pain and the interaction between both are still unclear. In this study, male Sprague–Dawley rats were used to study the modulation of NGF on orofacial pain through CCL19 and the role of each in tooth movement pain in rats. The expression levels of CCL19 mRNA and protein were determined by real-time PCR and immunofluorescence, respectively. Pain levels were assessed by measuring the rats' bite force, which drops as pain rises. Meanwhile, by verifying the relationship between CGRP and CCL19, it was laterally confirmed that NGF could modulate tooth movement-induced mechanical hyperalgesia through CCL19. The results showed that the expression level of CCL19 rose with the increased NGF, and neurons expressing CGRP can express stronger CCL19. Compared with the baseline level, the bite force for all rats dropped sharply on day 1, reached its lowest level on day 3, and recovered gradually on day 5. All results indicated that NGF played an important role in tooth movement orofacial pain *via* positively regulating CCL19 expression in the trigeminal ganglia of rats. Additionally, CCL19 increased the sensitivity to experimental tooth movement orofacial pain. NGF can regulate CCL19 expression, although it may regulate other inflammatory pathways as well. This is the first report on the interactions and modulations of tooth movement orofacial pain by NGF through CCL19 in rats.

## Introduction

Maxillofacial inflammatory pain caused by orthodontics presents as discomfort, dull pain, and hypersensitivity of the teeth. It is generated by the gradual tooth movement induced by orthodontic forces and the ensuing inflammatory reaction of periodontal tissue (1–3). In response to the periodontal inflammation, numerous leucocytes and inflammatory cells are recruited to and activated in periodontal tissues where they release abundant inflammatory mediators, chemokines, and cytokines including but not limited to nerve growth factor (NGF), tumor necrosis factor-alpha3, interleukin-1 (IL-1), IL-6, prostaglandins, interferon-gamma, and macrophage-colony stimulating factors, and ultimately result in tooth movement along with pain (4–8). Among these mediators, NGF is a target-derived neurotrophic factor that is mainly distributed within peripheral nerve tissue (9). It plays a critical role in the sensitization of sensory neurons to pain induced by peripheral nerve injury, especially in the trigeminal ganglion (TG) (10–12). In the course of the inflammatory reaction of periodontal tissue, activated mast cells release a massive amount of NGF that binds to tyrosine kinase A (TrkA). The NGF–TrkA complex is retrogradely transported to the TG and activates neurons as well as satellite glial cells (SGC) through interstitial links followed by upregulating NGF expression (13, 14). Elevated NGF augments maxillofacial pain by changing the expression of ion channels and neuropeptides in the TG (15, 16). Blocking NGF function by periodontal injection of anti-NGF antibody can completely abolish the orthodontic pain, whereas periodontal administrations of NGF can mimic the pain (17). All these findings indicate that NGF plays an important role in the pain regulation caused by tooth movement during orthodontic treatment.

In a previous study, we found that NGF participated in the modulation of orofacial pain (17). The ensuing periodontal tissue inflammation is accompanied by upregulated expression of a series of inflammatory mediators including CC chemokine ligand 19 (CCL19), chemokine (C-X-C motif) ligand 17 (CXCL17), CCL12, CXCL10, CXCL12, IL-15, CCL11, CXCL1, and IL-5 as identified both by isobaric tags for relative and absolute quantitation and by Bio-Plex bead microarray analysis. Among these meditators, CCL19 is expressed at the highest level (18). CCL19 is a small cytokine in the CCL chemokine family that is thought to maintain homeostasis in the internal environment of cells (19). It is usually involved in various inflammatory disorders and recognized as one of the pain-causing chemokines that promote neuroinflammatory responses and regulate pain by recruiting inflammatory cells after binding to its receptor, CCR7 (20, 21).

It is well-known that calcitonin gene-related peptide (CGRP) is evidenced to participate in the initiation and maintenance of inflammatory pain (22). Our previous study has demonstrated that CGRP participates in orthodontic pain following experimental tooth movement (6). Recent research has also proved that increased nociceptive neuropeptide expression is most certainly involved as NGF has been associated with the upregulation of CGRP, leading to neuroinflammatory pain response (23, 24). At present, relatively little is known about the relationship between CCL19 and NGF expression in oral and maxillofacial pain, and the role of NGF in combination with CCL19 in tooth movement pain is still unclear. However, if we can verify that CCL19 and CGRP have the same variation trend with NGF in tooth movement pain when exploring the relationship between CCL19 and NGF, we may hypothesize that NGF modulates tooth movement-induced mechanical hyperalgesia through CCL19. Therefore, in the current study, we extend our original observations in rats across multiple time points by inoculating NGF or anti-NGF antibody, NGF plus anti-CCL19 antibody, and anti-NGF antibody plus CCL19, respectively, to study the role of CCL19 in the regulation of orthodontic pain caused by NGF.

## Materials and Methods

### Animals

Two hundred and thirty-two male Sprague–Dawley rats with body mass of 200–250 g were obtained from the Animal Experimental Center at Sichuan University. They were maintained in the animal facility in an air-conditioned room at 21°C with a 12-h light–dark cycle. Standard rat chow and water were provided *ad libitum*. Animal experiments were performed in accordance with protocols that were approved by the Ethics Committee of the State Key Laboratory of Oral Diseases, Sichuan University (protocol no. WCHSIRB-D-2016-201).

As shown in Supplementary Figure 1, in our first study, to determine the effect of NGF injected into the TGs on CCL19 expression in the TGs after tooth movement, a total of 132 rats were used. Of these, 108 were randomly assigned to three groups of 36 each for the detection of CCL19 mRNA expression by quantitative polymerase chain reaction (qPCR): force + NGF (group 1), force + anti-NGF antibody (group 2), and force + normal saline (group 3). The remaining 24 rats were randomly divided into two groups of 12 each for the measurement of CCL19 protein expression by immunofluorescence: force + NGF (group 4) and force + normal saline (group 5). In the second study, to determine the functional role and contribution of elevated CCL19 levels on pain induced by tooth movement, 24 rats were randomly assigned to three groups of 8 rats each: force + CCL19 (group 6), force + anti-CCL19 antibody (group 7), and force + normal saline (group 8) (Supplementary Figure 2). In the third study, to investigate the effect of NGF on CCL19 expression following tooth movement, as displayed in Supplementary Figure 3, 40 rats were randomly assigned to five groups of 8 rats each: force + NGF (group 9), force + anti-NGF antibody (group 10), force + NGF + anti-CCL19 antibody (group 11), force + anti-NGF antibody + CCL19 (group 12), and force + normal saline (group 13). Moreover, to verify by immunofluorescence that NGF modulates tooth movement-induced mechanical hyperalgesia through CCL19, 36 rats were randomly divided into 6 groups: force + NGF (group 14), force + anti-NGF antibody (group 15), force + normal saline (group 16), pseudo-force + NGF (group 17), pseudo-force + anti-NGF antibody (group 18), and pseudo-force + normal saline (group 19) (Supplementary Figure 4). To induce tooth movement, every rat was anesthetized with intraperitoneal injection of 7% chloral hydrate in normal saline solution at 0.06 ml·g^−1^·body weight, after which a Ni–Ti alloy closed-coil spring was fixed in place with ligation wires (0.2 mm in diameter) between the left maxillary first molar and the upper incisor. The fixed spring in all force groups delivered a 40 g force as measured by a force meter (Xinya, Hangzhou, China) to simulate steady orthodontic forces inducing tooth movement, while a 0 g force was used for all the pseudo-force groups. Rats in groups 1–13 were euthanized by decapitation after being anesthetized with pentobarbital sodium (50 mg·kg^−1^·body weight) on days 0, 1, 3, 5, 7, and 14 (*n* = 6 per group per day), while rats in groups 14–19 were all sacrificed on day 3. Those rats that were euthanized on day 0 without receiving any interventions serve as the baseline controls for each study. All sections of this report adhere to the ARRIVE guidelines for reporting animal research (25).

### TG Injection

A microsyringe with a dose of 20 μl was used in trigeminal ganglion injection. The injection point was located at the midpoint of the line between the rat's left condylar and the posterior edge of the ascending ramus of the left mandible. The trigeminal ganglion was reached 9 mm after the injection needle was inserted at the injection site. Specific injection procedures have been reported in the previous literature (26).

For the study of NGF effects on CCL19 in the TGs, 10 μl aliquots of recombinant murine β-NGF (Peprotech, USA) and anti-NGF antibody (Abcam, UK) solutions at concentrations of 0.1 μg/μl in phosphate buffered saline (PBS, pH 7.1) for each were inoculated into the TG in groups 1 (force + NGF), 2 (force + anti-NGF antibody), and 3 (force + normal saline), respectively, on days 1, 3, 5, 7, and 14 following the experimental tooth movement. Meanwhile, animals in each group that were neither injected reagents nor had force on day 0 were used as the baseline controls. On each injection day, all rats in these three groups were injected, and six rats in each group were euthanized 6 h after inoculation on days 0, 1, 3, 5, 7, and 14. TGs were harvested and rapidly placed in liquid nitrogen for PCR and immunofluorescence assay. For the study of CCL19 effects on tooth movement-induced pain, the baseline bite force for all animals in groups 6 (force + CCL19), 7 (force + anti-CCL19 antibody), and 8 (force + normal saline) was measured before spring fixation. This was followed by inoculations into the TGs of 10 μl of 0.6 μg/μl recombinant rat CCL19 beta protein (R&D Systems, USA) in PBS, 10 μl of 0.6 μg/μl rat CCL19 beta antibody (R&D Systems, USA) in PBS, or 10 μl of normal saline solution on days 1, 3, 5, 7, and 14. For the study of the activation of CCL19 expression by NGF as well as the interactions between both, rats in groups 9 (force + NGF), 10 (anti-NGF antibody), 11 (force + NGF + anti-CCL19 antibody), 12 (force + anti-NGF antibody + CCL19), and 13 (force + normal saline) received TG inoculations of 10 μl of NGF solution, 10 μl of anti-NGF antibody solution, 10 μl of NGF solution plus 10 μl of anti-CCL19 antibody solution, 10 μl of anti-NGF antibody solution plus 10 μl of CCL19 solution, or 10 μl of normal saline, respectively, on days 1, 3, 5, 7, and 14 after a baseline of bite force testing on day 0 followed by applying the orthodontic force to induce tooth movement. It should be noted that the injection of either anti-CCL19 antibody in group 11 or CCL19 in group 12 was given 2 h after either NGF or anti-NGF antibody injection. Lastly, for the study of NGF regulating tooth movement pain through CCL19, rats in groups 14 (force + NGF), 15 (force + anti-NGF antibody), 16 (force + normal saline), 17 (pseudo-force + NGF), 18 (pseudo-force + anti-NGF antibody), and 19 (pseudo-force + normal saline) received TG injections of 10 μl of NGF solution, 10 μl of anti-NGF antibody solution, or 10 μl normal saline on days 1 and 3, respectively, after the establishment of the tooth movement model.

### Real-Time RT-PCR Assay

Total RNA was extracted from TGs using TaKaRa Minibest Universal RNA Extraction Kit (Takara, Shiga, Japan) according to the manufacturer's protocols. Then, cDNA was reverse transcribed using the PrimeScript^TM^ RT reagent kit with gDNA Eraser (Perfect Real Time) kit (TaKaRa, Shiga, Japan) following the manufacturer's recommendations.

Rat CCL19 mRNA expression was quantified in TG samples using triplex RT-PCR performed in a LightCycler480 (Roche, Switzerland) RT-PCR platform with TB Green^TM^ Premix Ex Taq (Perfect Real Time, TaKaRa, Dalian, China) according to the manufacturer's protocol. To quantify specific mRNA expression in the samples, a standard curve was generated for normal rat TG samples, and GAPDH served as an internal standard, using specific primers for rat GAPDH (forward primer CCAGCAAGGATACTGAGAGCAAG, reverse primer TGATGGTATTCGAGAGAAGGGAGG, expected size: 114 bp) and CCL19 (forward primer TAACGATGCGGAAGACTGCT, reverse primer CTGGTAGCCCCTTAGTGTGG, expected size: 139 bp). The final 20 μl reaction volume consisted of 10 μl of TB Green^TM^ Premix Ex Taq, 0.8 μl of the 10 μM primer mix, 4 μl of the reverse transcription nucleic acid template, and 5.2 μl of RNase-free H_2_O. The thermal profile was set at 95°C for 30 s and 95°C for 5 s, followed by 40 cycles at 60°C for 30 s and 72°C for 30 s. Relative mRNA transcript level calculations were performed using the comparative CT method (ΔΔCT). The average CT values of the three complex holes for each sample were calculated and marked as CT_CCL19_ and CT_GAPDH_, ΔΔCT = *N*(CT_CCL19_ – CT_GAPDH_) – (CT_CCL19_ – CT_GAPDH_).

### Immunofluorescence Assay

For CCL19 protein detection, TGs embedded in OCT were cut at 10 μm thickness along the TG long axis in a freezing microtome (Thermo Shandon, USA). The sections were rinsed with Tris-buffered saline Tween (TBST) three times for 5 min, underwent microwave-based antigen retrieval, and blocked with blocking buffer (Cell Signaling Technology, USA) for 60 min. Thereafter, the sections were stained with a primary mouse anti-CCL19/MIP-3 beta antibody (R&D Systems, USA) and incubated overnight at 45°C followed by rinsing with PBS (pH 7.1) three times for 5 min. The sections were further stained with an immunofluorescent rabbit anti-goat IgG secondary antibody (Bioss, Beijing, China), incubated for 1 h at 37°C, rinsed with PBS, and finally mounted onto slides for observation under a fluorescence microscope (Carl Zeiss, Goettingen, Germany). Images were taken by a Nikon Eclipse E400 microscope (Tokyo, Japan) and ImageJ 1.51 software was employed to analyze the average gray scale of each immunofluorescence image as the expression of CCL19 protein in the TG. The gray level of fluorescence immunoreactivity for CCL19 protein in the TG was taken as the mean of three measurements from three different areas of the slide.

Meanwhile, we used the same immunofluorescence protocols mentioned above to verify that the neurons expressing CGRP protein can also express CCL19 protein. The sections of TG tissues obtained from groups 14 to 19 were stained with primary rabbit anti-CGRP (D5R8F)/mAb antibody (Cell Signaling Technology, USA) and primary mouse anti-CCL19/MIP-3 beta antibody (R&D Systems, USA), respectively.

### Bite Force Measurement

The bite force of rats was determined following the protocols described previously (17). Briefly, after 1 week of acclimatization to the animal facility, rats were trained to bite the sensor (Nanjing, China) to obtain the baseline bite force. The next day, the spring to induce tooth movement was fixed in place. Thereafter, bite force was measured 6 h after inoculation of reagents into the TG on days 1, 3, 5, 7, and 14 following spring fixation, with triplicate measurements made for each rat. Changes in average bite force of triplicate measurements for each rat between prior and after inoculation interventions were recorded.

### Statistical Analyses

Statistical analyses were performed using SPSS 16.0 (SPSS, Chicago, Illinois, USA) and GraphPad Prism 7.0 software (GraphPad Software, San Diego, USA). The results are presented as mean ± standard deviation (SD). Student's *t*-test was used to compare the average gray scale of CGRP and CCL19 immunofluorescence in different groups at the same time point. A two-way ANOVA and a *post hoc* test were used to examine the effects of time (0, 1, 3, 5, 7, and 14 days) on the expression of CCL19 or bite force in the same group, the effects of different injections on the expression of CCL19 or bite force at the same time point, and the interactions with both time and the injection of reagents. For the two-group comparisons at each time interval, the Bonferroni *post hoc* test was used if the pretest for normality was not rejected at the 0.05 significance level. *p-*values < 0.05 were considered statistically significant.

## Results

### NGF Upregulates CCL19 Expression in Trigeminal Ganglia

**Figure 1:**
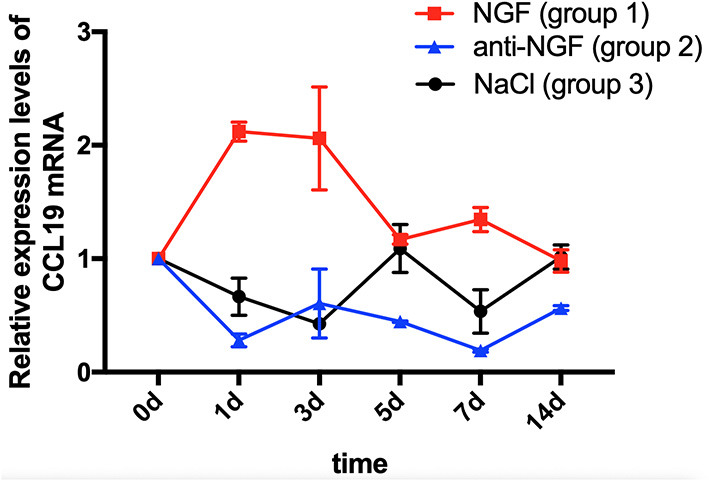
Time course of C-C chemokine ligand 19 (CCL19) mRNA expression in rats' TGs following the injection of nerve growth factor (NGF)-related reagents. (The statistical analysis results related to the image are shown in Table 1).

As displayed in Figure 1, a two-way ANOVA with repeated measures revealed that the expression levels of CCL19 mRNA were significantly influenced by group (*p* = 0.0021 < 0.01), time (*p* = 0.0121 < 0.05), and interactions (*p* = 0.0001 < 0.01). The level of CCL19 mRNA expression in rats' TG on day 0 (1.000 ± 0.000) obtained following the tooth movement pain model development was used as the baseline control. The mRNA expression level of CCL19 in rats injected with NGF increased sharply on day 1 (2.120 ± 0.085, *p* = 0.0001 < 0.001), remained at a high level on day 3 (2.060 ± 0.453, *p* = 0.0001 < 0.001), and decreased to the baseline level on day 5 (1.170 ± 0.042, *p* > 0.05), day 7 (1.345 ± 0.106, *p* > 0.05), and day 14 (0.980 ± 0.099, *p* > 0.05). By contrast, the mRNA expression level of CCL19 in rats' TG injected with anti-NGF antibody was significantly lower than the baseline level on the 1st day (0.280 ± 0.057, *p* = 0.0006 < 0.001), the 3rd day (0.605 ± 0.304, *p* = 0.0332 < 0.05), the 5th day (0.445 ± 0.007, *p* = 0.0042 < 0.01), the 7th day (0.190 ± 0.104, *p* = 0.0003 < 0.001), and the 14th day (0.565 ± 0.021, *p* = 0.0196 < 0.05). Meanwhile, the mRNA expression level of CCL19 in rats' TG in the control group injected with normal saline also showed a decreasing trend, falling on the 1st day (0.665 ± 0.163, *p* = 0.0736 > 0.05), reached to the lowest on the 3rd day (0.425 ± 0.007, *p* = 0.0033 < 0.01), briefly on the 5th day (1.090 ± 0.212, *p* = 0.9107 > 0.05), declined again on the 7th day (0.535 ± 0.191, *p* = 0.013, *p* < 0.05), and finally returned to the baseline level on the 14th day (1.015 ± 0.106, *p* = 0.9998 > 0.05).

Comparing CCL19 mRNA levels on days following NGF injections vs. normal saline injections, expression was significantly higher on days 1 (*p* = 0.0001 < 0.01), 3 (*p* = 0.0001 < 0.01), and 7 (*p* = 0.0002 < 0.01), but not different on days 5 and 14 (*p* > 0.05). On the other hand, comparing CCL19 mRNA levels on days following anti-NGF injections vs. normal saline injections, expression was significantly lower on days 5 (*p* = 0.0019 < 0.01) and 14 (*p* = 0.0276 < 0.05). Finally, comparing CCL19 mRNA levels on days following NGF injections vs. anti-NGF injections, expression was significantly higher on every injection day (*p* = 0.0001 < 0.05 for days 1–7, *p* = 0.0434 < 0.05 for day 14) (Table 1).

**Table 1:** *p*-value of C-C chemokine ligand 19 (CCL19) mRNA expression in rats' TGs following the injection of nerve growth factor (NGF)-related reagents.

**Time**	**Group 1 (NGF) vs**.	**Group 1 (NGF) vs**.	**Group 2 (anti-NGF) vs**.
**intervals**	**group 2 (anti-NGF)**	**group 3 (NaCl)**	**group 3 (NaCl)**
0 day	ns	ns	ns
1 day	<0.0001^****^	<0.0001^****^	ns
3 days	<0.0001^****^	<0.0001^****^	ns
5 days	0.0006^***^	ns	0.0019^**^
7 days	<0.0001^****^	0.0002^***^	ns
14 days	<0.0434^*^	ns	0.0276^*^

* *p < 0.05*,

** *p < 0.01*,

*** *p < 0.001*,

**** *p < 0.0001, ns: p > 0.05*.

*The variation tendency related to the statistical analysis results is shown in Figure 1*.

**Figure 2:**
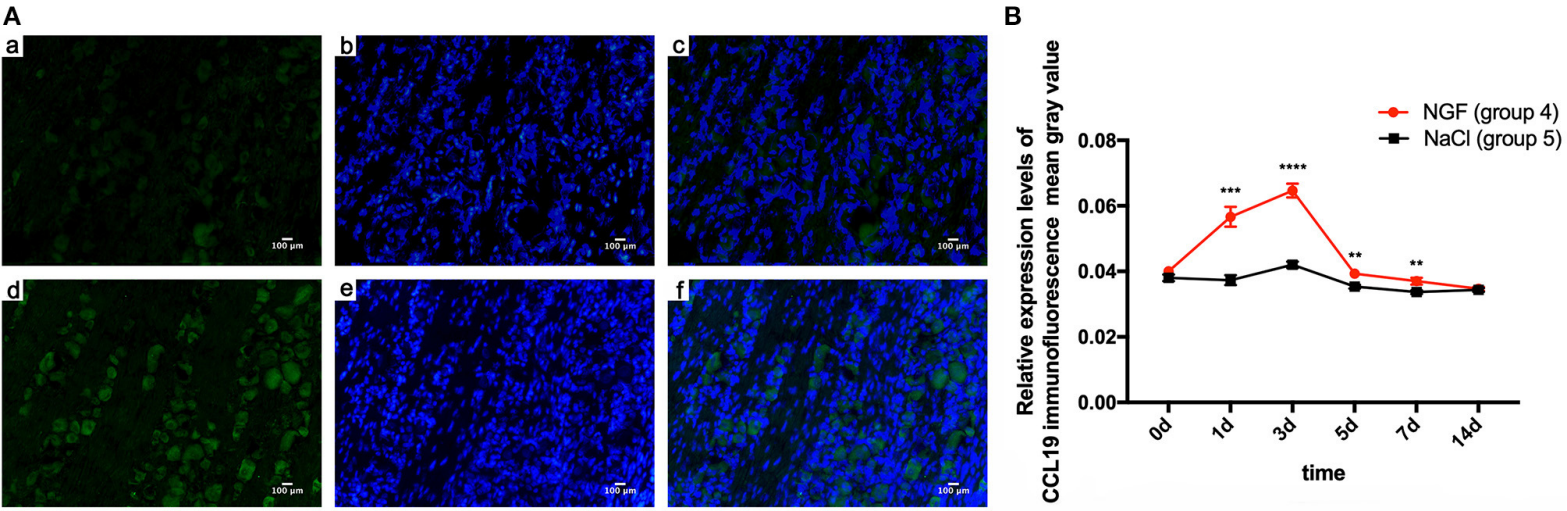
(**A**) Immunofluorescence results of CCL19 protein expression after injection of NGF or normal saline into rats' trigeminal ganglia (TGs) 3 days after initiation of tooth movement. (**a**) Neurons expressing CCL19 (green) after injection of normal saline on day 3 after initiation of tooth movement. (**b**) Nuclei (blue) after injection of normal saline on day 3. (**c**) Overlap of (**A**) and (**B**). (**d–f**) The same labeling as in (**a–c**) but after injection of NGF on day 3. (**B**) Time course of CCL19 protein expression in TGs following the injection of NGF or normal saline after experimental tooth movement. *p < 0.05, **p < 0.01, ***p < 0.001, ****p < 0.0001.

Immunofluorescent staining demonstrated the localization of CCL19 protein expression in TGs. Relative to the weak staining of CCL19 in group 5 (force + normal saline) saline-injected TGs, many CCL19-positive neurons were present in the TG in group 4 (force + NGF) NGF-injected TGs on days 1 and 3, with intensity being the highest on day 3 (Figure 2A). Two-way ANOVA revealed that CCL19 immunoreactivity in the TG was significantly influenced by group (*p* = 0.0001 < 0.01), time (*p* = 0.0001 < 0.01), and interaction (*p* = 0.0001 < 0.01).

Taking the mean gray value of immunofluorescence of CCL19 protein in rats' TG receiving NGF injection (group 4) on day 0 (0.040 ± 0.000) as the baseline, CCL19 immunoreactivity increased sharply on day 1 (0.057 ± 0.003, *p* = 0.0001 < 0.01), reached to its peak on day 3 (0.065 ± 0.002, *p* = 0.0001 < 0.01), then quickly declined on day 5 (0.039 ± 0.001, *p* = 0.9768 > 0.05), day 7 (0.037 ± 0.001, *p* = 0.0450 < 0.05), and day 14 (0.035 ± 0.001, *p* = 0.0003 < 0.001). On the other hand, the immunoreactivity of CCL19 protein in TG for saline-injected rats (group 5) was similar to the baseline on day 1 (0.037 ± 0.002, *p* = 0.9768 > 0.05), slightly increased on day 3 (0.042 ± 0.001, *p* = 0.0050 < 0.01), gradually decreased on days 5 (0.035 ± 0.001, *p* = 0.0891 > 0.05), and then dropped below baseline on day 7 (0.034 ± 0.001, *p* = 0.0024 < 0.01) and day 14 (0.034 ± 0.001, *p* = 0.0106 < 0.05). Further comparison analysis between group 4 and group 5 showed that the CCL19 protein fluorescence intensity in group 4 was significantly higher than that of group 5 on days 1 (*p* = 0.0006 < 0.001), 3 (*p* < 0.0001), 5 (*p* = 0.0058 < 0.01), and 7 (*p* = 0.0075 < 0.01) (Figure 2B). Hence, CCL19 protein in TGs with NGF inoculation was significantly higher than that with saline injection, consistent with the findings for CCL19 mRNA expression.

**Figure 3:**
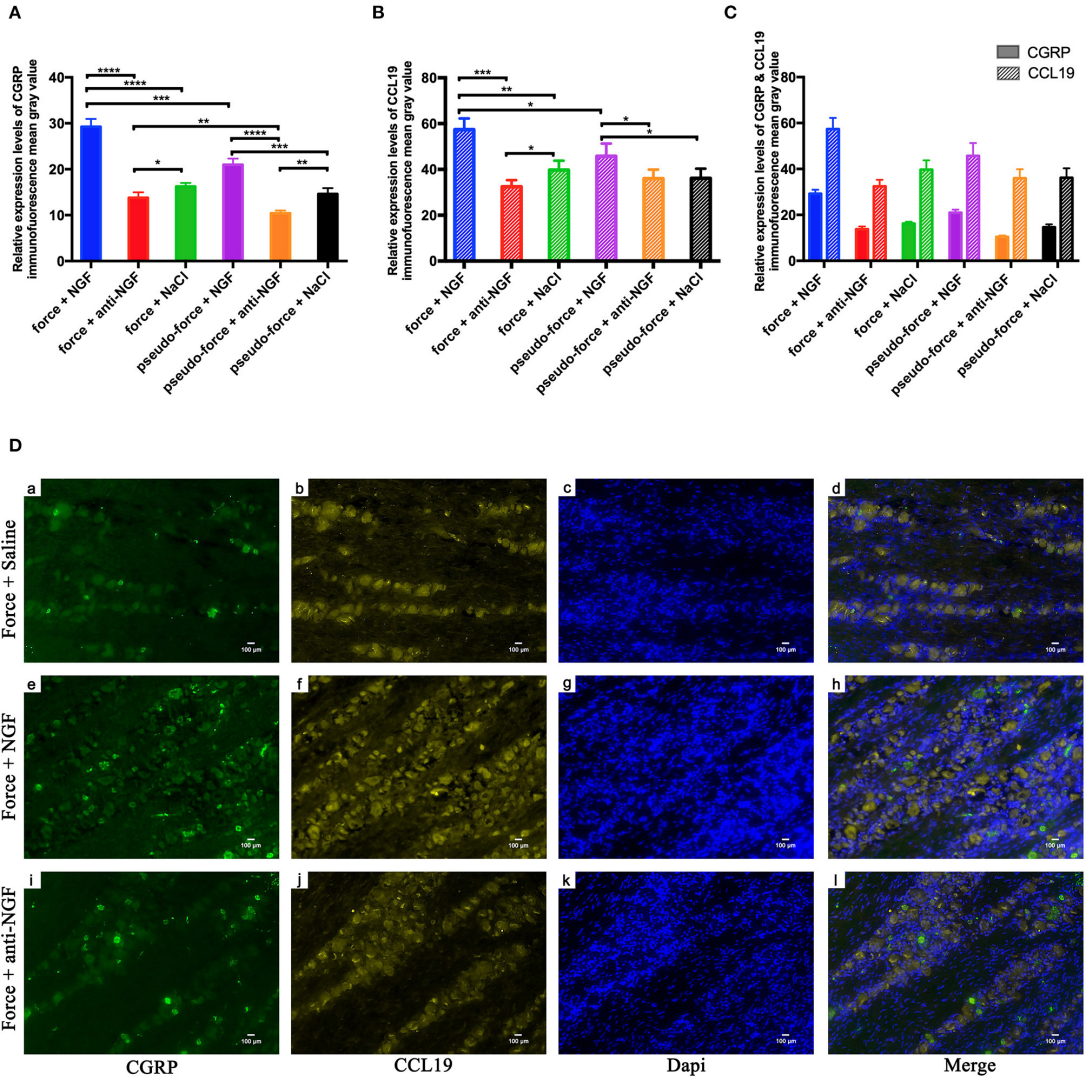
(**A**) Calcitonin gene-related peptide (CGRP) protein expression levels in TGs following different interventions after experimental tooth movement. (**B**) CCL19 protein expression levels in TGs following different interventions after experimental tooth movement. (**C**) Comparison of immunofluorescence mean gray value of CGRP and CCL19 relative expression. (**D**) Immunofluorescence results of protein expression after injection on NGF, anti-NGF antibody, and normal saline into forced rats' TGs on the 3rd day after tooth movement. (**a, e, i**) Neurons expressing CGRP (green) after injection of related reagents. (**b, f, j**) Neurons expressing CCL19 (yellow). (**c, g, k**) Nuclei (blue) after injection of related reagents on day 3. (**d, h, l**) Overlap of (**a + b + c**), (**e + f + g**), and (**i + j + k**). *p < 0.05, **p < 0.01, ***p < 0.001, ****p < 0.0001.

In our previous studies, we found that the orthodontic tooth movement-induced pain was most notable on the 3rd day following tooth movement (17, 27). Therefore, we conducted a further study on the expression of CGRP and CCL19 in the trigeminal ganglion of rats specifically on the 3rd day after tooth movement. As shown in Figure 3A, the expression level of CGRP was significantly higher in the force + NGF group (group 14) than in the force + anti-NGF group (group 15) and the force + saline group (group 16) (*p* < 0.0001). The expression of CGRP in group 15 and group 16 was also statistically significant (*p* = 0.0134 < 0.05). Immunoreactivity of CGRP protein in TGs for pseudo-forced rats in the pseudo-force + NGF group (group 17) was much higher than that of rats in the pseudo-force + anti-NGF group (group 18) (*p* < 0.0001) and the pseudo-force + saline group (group 19) (*p* = 0.0005 < 0.001), while the expression level of CGRP in group 18 was lower than that of rats in group 19 (*p* = 0.0011 < 0.01). In the meantime, the immunofluorescence protein expression of CGRP in rats in the forced group 14 was higher than that of rats in the pseudo-forced group 17 (*p* = 0.0003 < 0.001), as well as in group 15 and group 18 (*p* = 0.0026 < 0.01).

The trend of immunofluorescence expression of CCL19 in rats' trigeminal ganglia was similar to that of CGRP, as displayed in Figure 3B. The mean gray value of immunofluorescence observed in rats' trigeminal ganglia was the highest in group 14 among groups 14–19 (*p* < 0.0001 for group 14 vs. group 15, *p* = 0.0001 < 0.001 for group 14 vs. group 16, and *p* = 0.0201 < 0.05 for group 14 vs. group 17). Furthermore, the expression level of CCL19 immunofluorescence in forced rats' TGs injected with anti-NGF antibody in group 15 was significantly lower than that of rats injected with normal saline in group 16 (*p* = 0.0271 < 0.05). Similarly, rats in pseudo-forced group 17 with NGF injection expressed more CCL19 protein than the rats in group 18 (*p* = 0.0293 < 0.05) and group 19 (*p* = 0.0334 < 0.05).

One-way ANOVA showed that CGRP immunoreactivity in the TG of different groups on day 3 was statistical different among groups 14–19 (*p* = 0.0022 < 0.01) and the *p*-value of CCL19 protein expression was 0.0051 < 0.01. Meanwhile, using two-way ANOVA with repeated measures, it can be observed that the immunofluorescence expression levels were significantly influenced by groups (*p* < 0.0001) and target stain protein (*p* < 0.0001). It is worth noting that on the 3rd day, the mean gray value of immunofluorescence of CCL19 protein in trigeminal ganglion of rats was higher than that of CGRP protein in groups 14–19 (all *p* < 0.0001) (Figure 3C). It can be seen in Figure 3D that the neurons stained with CGRP also express CCL19. Meanwhile, it can also be observed that the expression intensity of CCL19 and CGRP in the trigeminal ganglion of rats injected with NGF was significantly higher than that of rats injected with anti-NGF antibody and normal saline.

### Effect of CCL19 in Rats' TG on Orofacial Pain Induced by Tooth Movement

Bite force was employed to assess the level of mechanical hyperalgesia in teeth as recorded by an occlusal force tester (Nanjing, China) and expected to decrease to the degree when biting induces pain. A two-way repeated measures ANOVA showed that bite force in all groups was significantly influenced by group (*p* = 0.0001 < 0.01), time (*p* = 0.0001 < 0.01), and interactions (*p* = 0.0144 < 0.05).

**Figure 4:**
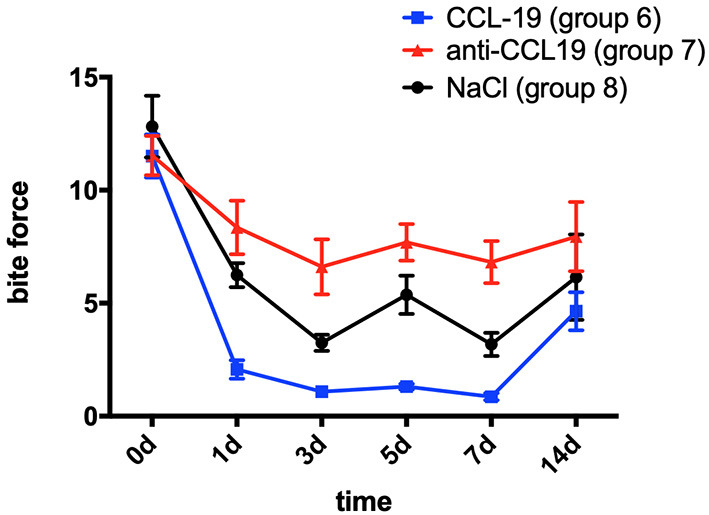
Changes in pain expressed as the bite force of rats over time after injection of CCL19 and anti-CCL19 antibody. (The statistical analysis results related to the image are shown in Table 2).

In comparison with the baseline level measured on day 0 (11.512 ± 2.692), the bite force of rats with CCL19 inoculation (group 6) plummeted on day 1 (2.069 ± 1.155, *p* < 0.0001) and continued to decrease on day 3 (1.086 ± 0.658, *p* < 0.0001), slightly rose on day 5 (1.308 ± 0.366, *p* < 0.0001), reached its lowest level on day 7 (0.864 ± 0.445, *p* < 0.0001), and then recovered but still lower than the baseline on day 14 (4.463 ± 2.375, *p* < 0.0001) (Figure 4). Regarding the bite force of rats in group 7 (force + anti-CCL19 antibody), it dropped sharply on the 1st day (8.351 ± 3.360, *p* = 0.0482 < 0.0001), declined to its lowest level on the 3rd day (6.608 ± 3.435, *p* = 0.0006 < 0.01), increased slightly on the 5th day (7.689 ± 2.288, *p* = 0.0111 < 0.05), decreased a little on the 7th day (6.819 ± 2.638, *p* = 0.0012 < 0.01), and gradually raised to near the baseline on the 14th day (7.950 ± 4.334, *p* = 0.0203 < 0.05) in comparison with the baseline of bite force measured on day 0 (11.512 ± 2.460). In the meantime, the bite force of rats in group 8 (force + normal saline) significantly decreased on day 1 (6.244 ± 1.501, *p* < 0.0001), dropped to the lowest on day 3 (3.249 ± 1.016, *p* < 0.0001), increased slightly on day 5 (5.371 ± 2.403, *p* < 0.0001), continued to decrease on day 7 (3.179 ± 1.443, *p* < 0.0001), and then rose but still below the baseline on day 14 (6.152 ± 5.351, *p* < 0.0001), compared with those of baseline on day 0 (12.815 ± 3.863). Representative bite patterns are shown in Supplementary Figure 5.

Rats with anti-CCL19 antibody inoculation (group 7) shared similar trends of bite force with rats in group 6 but at a much higher level (*p* < 0.0001 for days 1, 5, and 7, respectively, *p* = 0.0002 < 0.05 for day 3, and *p* = 0.0399 < 0.05 for day 14). Additionally, as displayed in Figure 4, the bite force in group 6 was significantly lower than that of the saline controls (group 8) on day 1 (*p* = 0.0065 < 0.01) and day 5 (*p* = 0.0084 < 0.01). Therefore, the lowest bite force following tooth movement was found in rats inoculated with CCL19, while the highest one was found in rats with anti-CCL19 antibody inoculation. It is noteworthy that elevated bite force was seen in rats injected with anti-CCL19 antibody as compared with saline on day 3 (*p* = 0.0361 < 0.05) and day 7 (*p* = 0.0208 < 0.05). All these results illustrate that CCL19 has a positive sensitization to experimental tooth movement pain in rats. Details of significant statistical differences are shown in Table 2.

**Table 2:** *p*-value of bite force of rats over time after injection of CCL19 and anti-CCL19 antibody.

**Time**	**Group 6 (CCL19) vs**.	**Group 6 (CCL19) vs**.	**Group 7 (anti-CCL19) vs**.
**intervals**	**group 7 (anti-CCL19)**	**group 8 (NaCl)**	**group 8 (NaCl)**
0 day	ns	ns	ns
1 day	<0.0001^****^	0.0065^**^	ns
3 days	0.0002^***^	ns	0.0361^*^
5 days	<0.0001^****^	0.0084^**^	ns
7 days	<0.0001^****^	ns	0.0208^*^
14 days	0.0399^*^	ns	ns

* *p < 0.05*,

** *p < 0.01*,

*** *p < 0.001*,

**** *p < 0.0001, ns: p > 0.05*.

*The variation tendency related to the statistical analysis results is shown in Figure 4*.

### Regulation of CCL19 Expression and Its Interaction With NGF Following Tooth Movement

**Figure 5:**
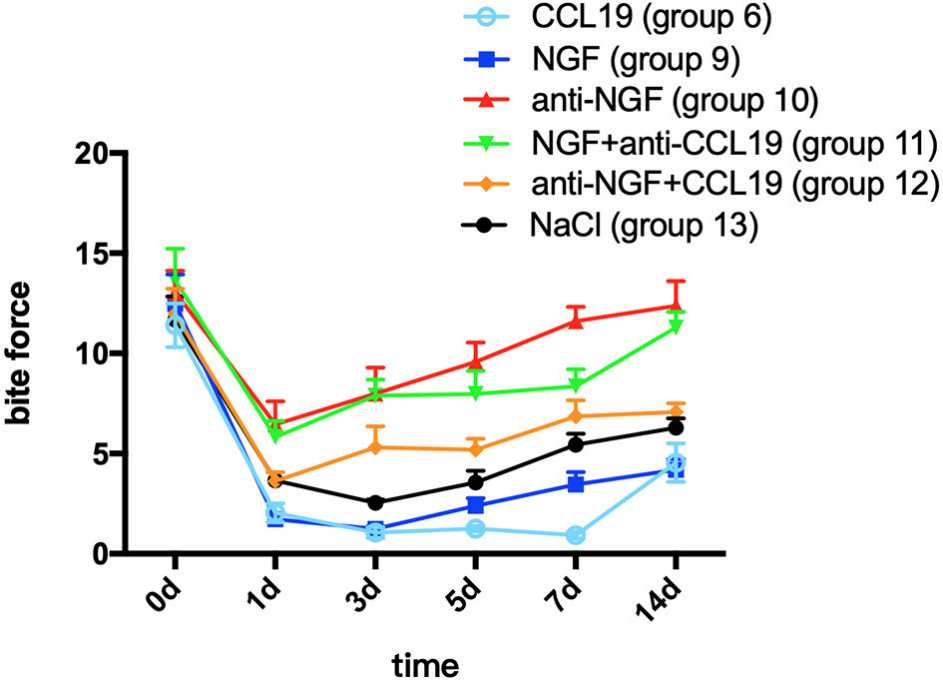
Changes in pain expressed as the bite force modulated by NGF *via* CCL19 over time. (The statistical analysis results related to the image are shown in Table 3).

A two-way repeated measures ANOVA analysis showed that bite force in groups 9 (force + NGF), 10 (force + anti-NGF antibody), 11 (force + NGF + anti-CCL19 antibody), 12 (force + anti-NGF antibody + CCL19), and 13 (force + normal saline) was significantly influenced by group (*p* < 0.0001), time (*p* < 0.0001), and interactions (*p* = 0.0016 < 0.01). In comparison with baseline bite force measured before spring fixation on day 0, the bite force of rats in group 9 with NGF inoculation decreased sharply on day 1 (1.719 ± 0.816, *p* = 0.0001 < 0.01), declined to a minimum on day 3 (1.248 ± 0.364, *p* = 0.0001 < 0.01), and slightly increased on day 5 (2.390 ± 1.107, *p* = 0.0001 < 0.01), day 7 (3.466 ± 1.740, *p* = 0.0001 < 0.01), and day 14 (4.194 ± 1.495, *p* = 0.0001 < 0.01) although still well-below baseline. As shown in Figure 5, similar trends in bite force were observed across study days for rats injected with anti-NGF (group 10), NGF + anti-CCL19 (group 11), and anti-NGF antibody + CCL19 (group 12). Compared with the baseline on day 0 (13.054 ± 3.052), the bite force of rats injected with anti-NGF (group 10) dropped to its lowest value on the 1st day (6.459 ± 3.234, *p* < 0.0001), increased slightly on the 3rd day (7.997 ± 3.664, *p* = 0.0002 < 0.001) and the 5th day (9.568 ± 2.731, *p* = 0.0252 < 0.05), and returned to the baseline level on the 7th day (11.607 ± 2.021, *p* = 0.7869 > 0.05) and the 14th day (12.372 ± 3.500, *p* = 0.9901 > 0.05). For rats injected with NGF + anti-CCL19 antibody in group 11, the bite force measured on each time interval was 5.820 ± 2.254 (*p* < 0.0001) for the 1st day, 7.894 ± 2.243 (*p* < 0.0001) for the 3rd day, 7.971 ± 3.286 (*p* < 0.0001) for the 5th day, 8.364 ± 2.388 (*p* < 0.0001) for the 7th day, and 11.287 ± 2.216 (*p* = 0.2853 > 0.05) for the 14th day, respectively, in comparison with the baseline level of 13.646 ± 4.472 obtained on day 0. For rats injected with anti-NGF antibody + CCL19 in group 12, the bite force decreased sharply on day 1 (3.631 ± 1.271, *p* < 0.0001) and continued to increase on day 3 (5.309 ± 2.972, *p* < 0.0001), day 5 (5.191 ± 1.550, *p* < 0.0001), day 7 (6.860 ± 2.252, *p* = 0.0001 < 0.001), and day 14 (7.081 ± 1.199, *p* = 0.0003 < 0.001) but still well-below the baseline level of 11.971 ± 3.549 measured on day 0.

In terms of the regulation of CCL19 expression by NGF in experimental tooth movement pain, a two-way repeated measures ANOVA revealed that the bite force of rats injected with NGF alone (group 9) was much lower than that of rats injected with NGF + anti-CCL19 (group 11) on day 1 (*p* = 0.0096 < 0.01), day 3 (*p* = 0.0001 < 0.01), day 5 (*p* = 0.0001 < 0.01), day 7 (*p* = 0.0010 < 0.01), and day 14 (*p* = 0.0001 < 0.01). Meanwhile, the bite force of rats injected with anti-NGF alone (group 10) was significantly higher than that of rats injected with anti-NGF antibody + CCL19 (group 12) on day 5 (*p* = 0.0046 < 0.01), day 7 (*p* = 0.0016 < 0.01), and day 14 (*p* = 0.0003 < 0.01). In addition, it can be found that the bite force of rats injected with anti-NGF antibody + CCL19 (group 12) was much higher than that of rats injected with CCL19 alone (group 6) on day 1 (*p* = 0.0222 < 0.05), day 3 (*p* = 0.0015 < 0.01), day 5 (*p* < 0.0001), day 7 (*p* < 0.0001), and day 14 (*p* = 0.0213 < 0.05), respectively (Table 3). Also, as shown in Figure 5, rats injected with NGF (group 9) and rats injected with CCL19 (group 6) both expressed low level bite force measurement values, but there was no statistical difference between these two groups. Therefore, NGF, at least but not unique, is involved in the pain signaling pathways activated by orthodontic treatment *via* regulation of CCL19 expression.

**Table 3:** *p*-value of bite force of rats over time after injection of CCL19/NGF-related reagents.

**Time intervals**	**Group 9 (NGF) vs**.	**Group 10 (anti-NGF) vs**.	**Group 6 (CCL19) vs**.
	**group 11 (NGF + anti-CCL19)**	**group 12 (anti-NGF + CCL19)**	**group 12 (anti-NGF + CCL19)**
0 day	ns	ns	ns
1 day	0.0096^**^	ns	0.0222^*^
3 days	<0.0001^****^	ns	0.0015^**^
5 days	0.0001^***^	0.0046^**^	<0.0001^****^
7 days	0.0010^***^	0.0016^**^	<0.0001^****^
14 days	<0.0001^****^	0.0003^***^	0.0213^*^

* *p < 0.05*,

** *p < 0.01*,

*** *p < 0.001*,

**** *p < 0.0001, ns: p > 0.05*.

*The variation tendency related to the statistical analysis results is shown in Figure 5*.

## Discussion

Orthodontic pain arising from the periodontal inflammatory responses induced by orthodontic forces is a very common complaint. Previous studies have shown that the trigeminal ganglion can produce a large number of inflammatory factors following tooth movement pain (28, 29). These inflammatory factors alter the biological activity of the neurons in the trigeminal ganglia, increasing excitability of neurons (30, 31), leading to thermal and mechanical sensitivity to pain (32, 33). Increased expression of chemokines including CCL2, CXCL9, and CXCL10 is of particular interest because they promote synovial inflammation by stimulating leukocyte migration and are known to be involved in orofacial neuropathic pain (34, 35). A positive correlation between their concentration in the TGs and orofacial pain induced by orthodontic treatment has been reported (36). To our best knowledge, this is the first report of CCL19 expression and regulation as well as its functional role in tooth movement-induced pain generation in a rat model.

CCL19, also known as macrophage inflammatory protein-3β and EBI-1 ligand chemokine, is a small cytokine belonging to the C-C chemokine family. Increased expression of CCL19 can promote inflammatory responses (37). Our data emphasize that inoculation of CCL19 into the TG after spring fixation aggravates tooth movement-induced pain, whereas the anti-CCL19 antibody can reduce the pain. Specifically, elevation of CCL19 mRNA and protein levels following spring fixation-induced tooth movement was significantly associated with increased pain as reflected in reduced bite force, while injections of anti-CCL19 provided pain relief. Thus, CCL19 plays a crucial role in nervous system sensitization in inflammation-associated tooth movement-induced orofacial pain.

As a protein involved in the growth of neurons and the repair of nerve tissues, NGF plays an important role in pain regulation. The expression of this protein can be upregulated by damage to peripheral axons and retrogradely transported to the central processes of sensory neurons (13). Specifically, in the process of experimental tooth movement in rats, trigeminal nerve endings of the periodontal ligament were damaged, followed by the upregulation of NGF expression in rats' periodontal membrane (38). The signals were then transmitted in reverse to the trigeminal ganglia, which activated the neurons located in the trigeminal ganglia. The neurons then activated the satellite glial cells and led to upregulated NGF expression in TG (14). NGF mRNA and protein are expressed in the neurons and satellite glial cells of the TGs as can be seen with *in situ* hybridization and immunocytochemistry, respectively.

There are many biological mechanisms of NGF-induced trunk mechanical hyperalgesia. Recent studies have shown that NGF is related to the upregulation of CGRP in dorsal root ganglion and spinal dorsal horn (23). CGRP is a well-known contributor to persistent neurogenic inflammation (22). Activation of CGRP receptors located at the terminals of primary afferent neurons can reduce the activation threshold of secondary neurons, increasing the synaptic strength between nociceptors and spinal dorsal horn neurons, which ultimately promote mechanical and thermal sensitization. Eventually, these changes in intracellular signaling pathways contribute to persistent hyperalgesia. Therefore, by studying the relationship between CCL19 and CGRP, the interaction between CCL19 and NGF in tooth movement pain in rats can be laterally verified.

Our previous study found that NGF may regulate tooth movement pain in the trigeminal ganglia through the pro-neuroinflammatory response pathway (18). The expression of multiple inflammatory mediators was upregulated by NGF-induced tooth movement pain. Regarding the interactions between NGF and CCL19 expression, pairwise comparison showed that expression of CCL19 mRNA and protein in rats in the presence of NGF is significantly higher than in controls, while the expression of CCL19 mRNA and protein in rats with anti-NGF inoculation was much lower than in controls. Similarly, our published data also indicate that rats show a significantly reduced “functional status” of bite force due to tooth movement pain compared with their controls (17). In this experiment, we also found that the expression levels of CCL19 in the force groups were higher than those in the pseudo-force groups. Meanwhile, after the injection of NGF and CCL19, respectively, the corresponding rats both showed extremely low level of bite force measurement values. All these results suggest that CCL19 is specifically linked to orofacial pain induced by tooth movement. In addition to the above research findings, the experimental results of groups 14–19 showed that the immunofluorescence expression intensity of CCL19 changed with the injection of NGF-related reagents. Besides, CCL19 can be expressed in the trigeminal ganglion neurons of rats with CGRP expression, and the immunoactivity of the former was stronger than that of the latter, indicating that NGF can modulate tooth movement-induced mechanical hyperalgesia through CCL19. These results further validate our hypothesis that the increased NGF can significantly upregulate the expression of inflammatory mediator CCL19 in rats' TG. The increased CCL19 expression further facilitates the response to neuroinflammation and releases more inflammatory factors, amplifying and augmenting the pain induced by tooth movement. Therefore, neutralization of NGF or CCL19 will be an option for the relief of pain induced by orthodontic tooth movement.

Moreover, rats injected with NGF + anti-CCL19 antibody (group 11) present significantly higher bite force than those injected with NGF alone (group 9). This indicates that tooth movement pain sensitized by NGF can be alleviated by blocking CCL19. Thus, we can suppose that CCL19 is a downstream signaling pathway of NGF and NGF regulates tooth movement pain through CCL19. Meanwhile, CCL19 may also be regulated by other inflammatory factors that affect tooth movement pain. This hypothesis was proposed owing to the fact that the bite force of rats injected with NGF + anti-CCL19 antibody should be consistent with that of rats injected with normal saline because the antibody against CCL19 neutralized the pain caused by NGF, if CCL19 is only regulated by NGF. Actual results contrary to the above speculation obtained in the present study suggest that the expression of CCL19 may also be regulated by other inflammatory factors other than NGF. Similarly, by comparing the bite force of rats injected with anti-NGF antibody (group 10), anti-NGF antibody + CCL19 (group 12), and NS (group 13), we can conclude that the modulation of tooth movement pain by NGF may also be through other pathways besides CCL19.

Limitations of this study include the lack of *in vitro* study with cells. Further studies are needed to determine the relationship between the expression of this chemokine with the postoperative prognosis of orthodontic treatment, as well as to determine whether NGF regulates not only CCL19 but also other related molecular pathways that mediate tooth movement pain.

In conclusion, this study investigated the molecular mechanism of pain induced by tooth movement based on a rat model. Firstly, CCL19 expression levels in rats' TGs were studied by qPCR and immunofluorescence. It was confirmed that NGF positively regulated CCL19 expressions. Secondly, CCL19 in TGs plays an important role in tooth movement-induced orofacial pain in rats measured by bite force, which can objectively reflect the changes of pain levels. Finally, we continue to use this test as an indicator to elucidate the modulation mechanisms of experimental tooth movement orofacial pain by NGF through CCL19 pathway in rats.

## Data Availability Statement

All datasets generated for this study are included in the article/Supplementary Material.

## Ethics Statement

The animal study was reviewed and approved by the ethical committee of the state key laboratory of oral diseases, Sichuan University.

## Author Contributions

RG, JW, HY, YZ, MG, and HLi carried out the experiment. RG, YC, and HLo analyzed the data, discussed the results, and drafted the manuscript in consultation with WL. HLo offered domain knowledge in the design of the system. WL conceived the study and was in charge of overall direction and planning. RG, LL, HLo, and WL critically revised and commented on the manuscript. All authors contributed to the article and approved the submitted version.

## Conflict of Interest

The authors declare that the research was conducted in the absence of any commercial or financial relationships that could be construed as a potential conflict of interest.
