# Supplementary material for: Nerve Growth Factor Enhances Tooth Mechanical Hyperalgesia Through C-C Chemokine Ligand 19 in Rats
Source: Front Neurol. 2021 Jun 1;12:540660. doi: 10.3389/fneur.2021.540660 (PMC8211465; doi:10.3389/fneur.2021.540660)
Supplement: Supplementary file 1 [file Data_Sheet_1.PDF]

Supplementary figure legends

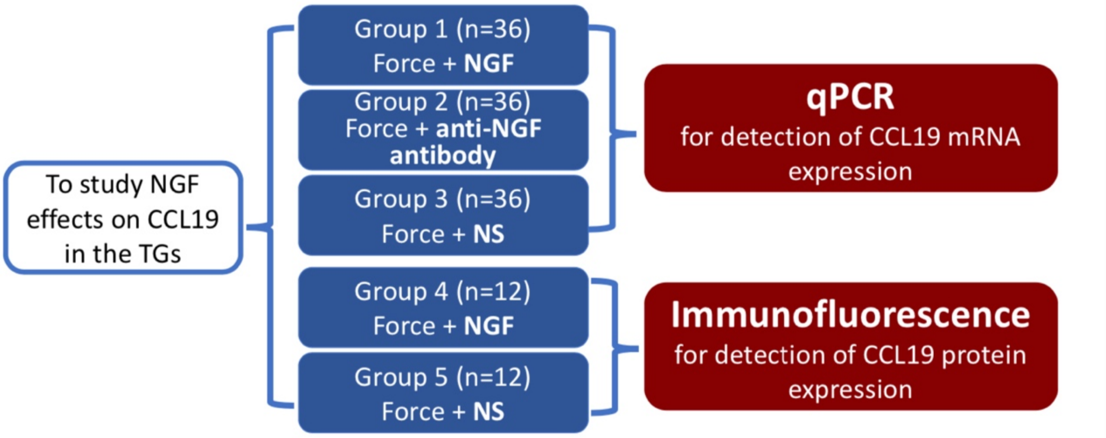

Supplementary figure 1. Experimental design for NGF upregulating CCL19 expression in trigeminal ganglia.

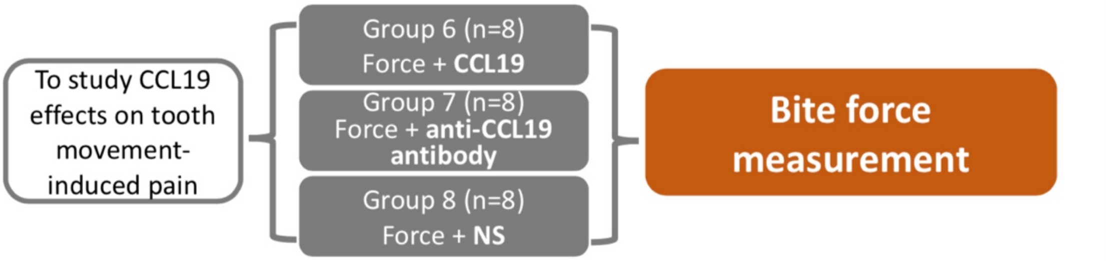

Supplementary figure 2. Experimental steps to detect the effect of CCL19 in rats' TG on orofacial pain induced by tooth movement

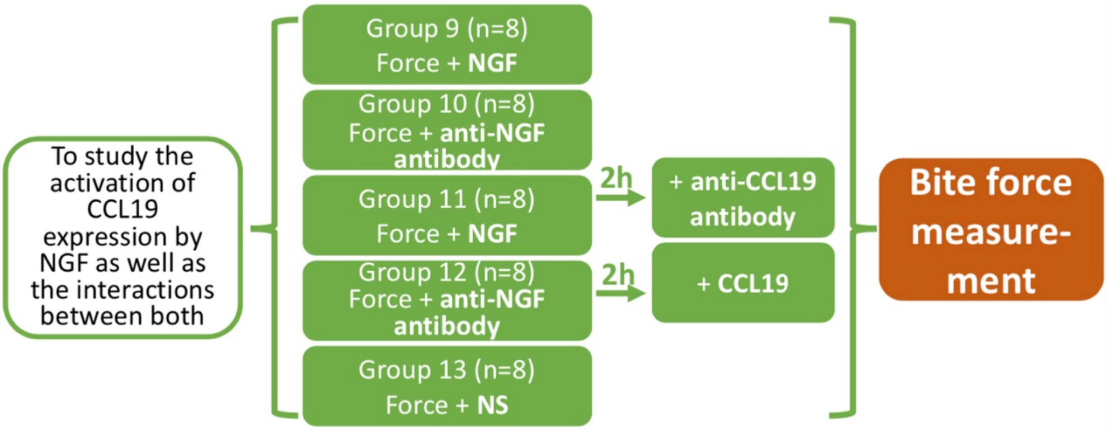

Supplementary figure 3. Experimental protocol to detect regulation of CCL19 expression and its interaction with NGF following tooth movement

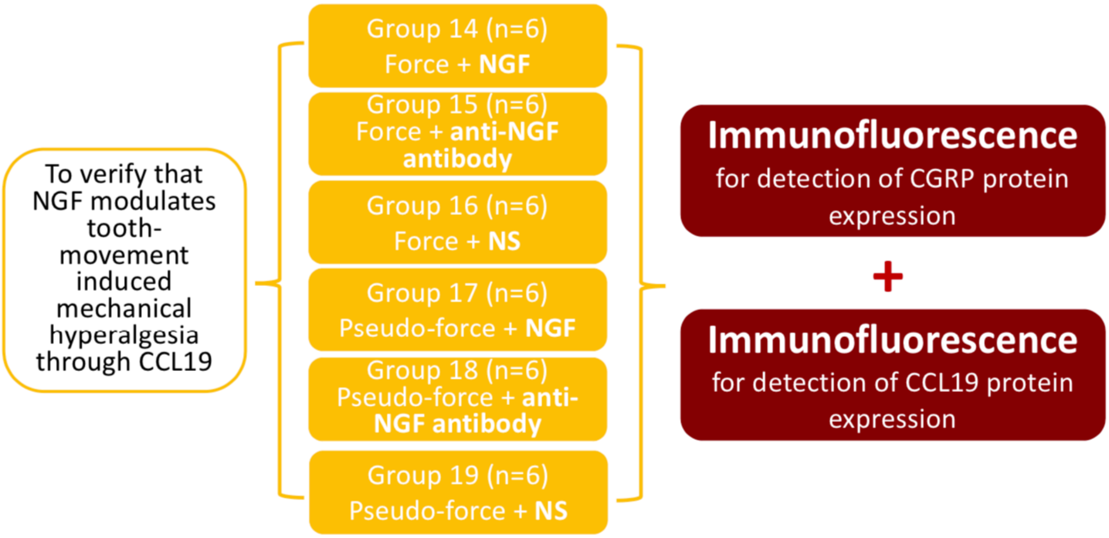

Supplementary figure 4. Experimental procedures to study verify that NGF causes tooth movement pain through CCL19 in trigeminal ganglia.

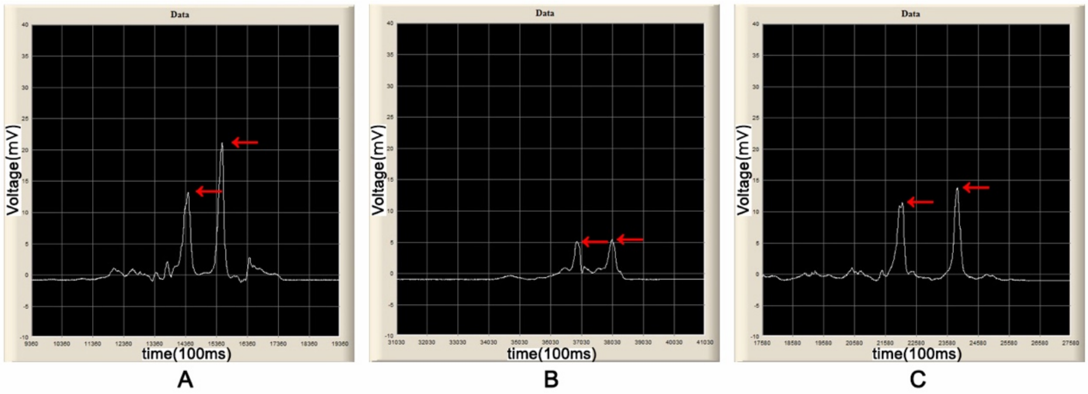

Supplementary figure 5. Representative bite patterns of rats during tooth movement. (A) bite pattern on day 0; (B) bite pattern on day 3; (C) bite pattern on day 7.
